# A 100 KS/s 8–10-Bit Resolution-Reconfigurable SAR ADC for Biosensor Applications †

**DOI:** 10.3390/mi13111909

**Published:** 2022-11-05

**Authors:** Yunfeng Hu, Lexing Hu, Bin Tang, Bin Li, Zhaohui Wu, Xiaojia Liu

**Affiliations:** 1 University of Electronic Science and Technology of China, Zhongshan Institute, Zhongshan 528402, China; 2 School of Microelectronics, South China University of Technology, Guangzhou 510640, China

**Keywords:** SAR ADC, resolution-reconfigurable, switching scheme

## Abstract

A DAC switching scheme that combines energy efficiency and resolution reconfigurability is proposed. Compared with the conventional switching scheme, the proposed scheme achieves 93.8%, 96.1%, and 97.3% switching energy saving in 8-bit, 9-bit, and 10-bit modes, respectively. Based on the proposed switching scheme, an 8–10-bit resolution-reconfigurable SAR ADC for biosensor applications is designed. The ADC consists of resolution-reconfigurable binary-weighted capacitive DAC, a two-stage full dynamic comparator, sampling switch, and the resolution-control SAR logic. Simulated in 180 nm CMOS process and 100 kS/s sampling rate, the ADC achieves the 46.80/53.89/60.14 dB signal-to-noise and distortion ratio (SNDR), the 55.22/62.51/73.09 dB spurious-free dynamic range (SFDR) and the 0.81/0.91/1.01 μW power consumption in 8/9/10-bit mode, respectively.

## 1. Introduction

In recent years, successive-approximation register (SAR) analogue-to-digital converters (ADCs) have been preferred for biosensor applications [1,2,3,4,5]. SAR ADC is based on a successive approximation algorithm, which is suitable for designing resolution-reconfigurable SAR ADCs [6,7,8]. Resolution-reconfigurable ADCs can choose the appropriate resolution according to the characteristics of the biomedical signal, thus reducing energy consumption. Recently, several energy-efficient DAC switching schemes have been developed to improve the power efficiency of DAC capacitor arrays [9,10,11]. Compared to a conventional switching scheme [12], capacitor splitting [9], set and down [10], and V_cm_-based [11] reduce the switching energy by 37.4%, 81.3%, and 87.5%, respectively. However, their switching energy doubles as the number of bits increases by one. Taking the V_cm_-based switching scheme as an example, the average switching energy of 8-bit, 9-bit and 10-bit is 42.2 $CV_{ref}^{2}$, 84.8 $CV_{ref}^{2}$ and 170.2 $CV_{ref}^{2}$, respectively.

In this paper, a DAC switching scheme that combines energy efficiency and resolution reconfigurability is proposed. The average switching energy of the proposed switching scheme for 8-bit, 9-bit, and 10-bit SAR ADCs is 21.2 $CV_{ref}^{2}$, 26.5 $CV_{ref}^{2}$, and 37.1 $CV_{ref}^{2}$, which amounts to a reduction of 93.8%, 96.1% and 97.3% in the switching energy compared with a conventional switching scheme [9]. Based on the proposed switching scheme, a 100 KS/s 8–10-bit resolution-reconfigurable SAR ADC for biosensor applications is designed and simulated. In order to reduce the on-resistance of the sampling switch and reduce the sampling error, the sampling switch adopts a bootstrap switch circuit [13]. A two-stage full dynamic comparator is used to achieve low power consumption [14]. To improve the performance of SAR logic, a dynamic logic unit is used [15]. Based on the SAR logic, we design a resolution control circuit for bit control. When the SAR ADC is operating in 8-bit or 9-bit resolutions, in order to save power, two or one dynamic logic units need to be skipped, respectively. The frequency range of various biomedical signals are shown in Table 1. The 100KS/s sampling rate can meet these biomedical signal sensing applications.

This paper is organized as follows: Section 2 describes the ADC architecture and presents the techniques used to achieve resolution reconfigurability and low power; the simulated results and the comparison with the state of the art are provided in Section 3. The conclusion is drawn in Section 4.

## 2. Proposed SAR ADC Architecture

**Figure 1:**
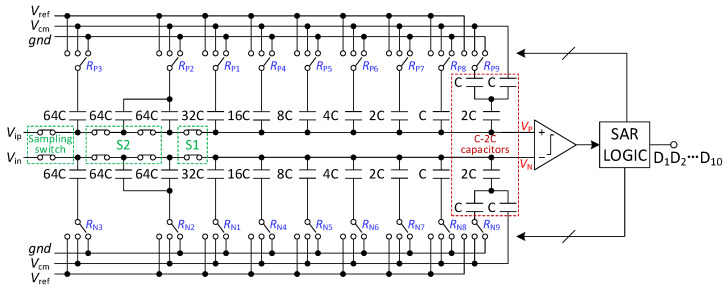
Architecture of 8–10-bit resolution-reconfigurable SAR ADC.

Figure 1 shows the architecture of the proposed 8–10-bit resolution-reconfigurable SAR ADC. It comprises a resolution-reconfigurable binary-weighted capacitive DAC, a two-stage full dynamic comparator, sampling switch, and the resolution-control SAR logic.

### 2.1. Proposed DAC Switching Scheme

The proposed DAC switching scheme is similar to a *V*_cm_-based switching scheme [11]. However, the proposed DAC has replaced the dummy capacitor with the C-2C capacitors, which add one bit of accuracy, and adds some switches (S1 and S2) to adjust the resolution. As shown in Table 2, resolutions of ADC are adjusted by S1 and S2. In the 8-bit mode, S1 and S2 are always opened (except in the sampling phase). For the 9-bit mode, S1 will be closed in the 3rd comparison, with S2 remaining open. For the 10-bit mode, S1 will be closed in the 3rd comparison and S2 will be closed in the 4th comparison. A special reference switching method, which is shown in Table 3 and Table 4, is used for the references (*R*_P2_, *R*_N2_, *R*_P3_ and *R*_N3_) associated with switches S1 and S2. Table 3 shows the references of *R*_P2_ and *R*_N2_ for each phase in 9-bit and 10-bit modes. The references of *R*_P2_ and *R*_N2_ are determined by the results of the 1st comparison (*D*_1_) and the 2nd comparison (*D*_2_). Table 4 shows the references of *R*_P3_ and *R*_N3_ for each phase in 10-bit mode. *R*_P3_ and *R*_N3_ are determined by the results of the first comparison (*D*_1_) and the 3rd comparison (*D*_3_).

To explain the operation of the SAR ADC, the proposed switching scheme in the three modes is used as follows.

#### 2.1.1. Proposed 8-Bit Mode Switching Scheme

**Figure 2:**
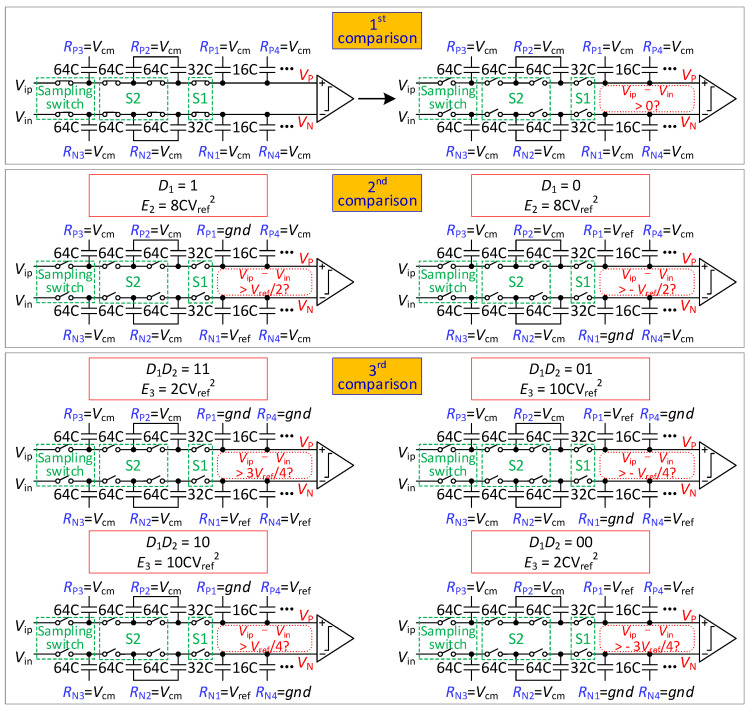
Proposed 8-bit mode switching scheme.

The steps of the conversion process of 8-bit mode are illustrated in Figure 2.

Initially, in the sampling phase, all switches are closed, and the reference voltage of all capacitors is set to *V*_cm_. The input voltage is sampled onto the top plates of the capacitors. 

In the 1st comparison, all switches are opened, and the output voltage of the capacitor array is found to be

(1) $$\left\{ \begin{array}{l} V_{P}\left(1\right)=V_{ip}\\ V_{N}\left(1\right)=V_{in} \end{array}\right.$$

The comparator compares the sampling signals (*V_ip_* and *V_in_*) and obtains *D*_1_.

In the 2nd comparison, if *D*_1_ is 1, the reference voltage of *R*_P1_ is changed from *V*_cm_ to *gnd*, and *R*_N1_ is changed from *V*_cm_ to *V_ref_*.. If *D*_1_ is 0, the reference voltage of *R*_P1_ is changed from *V*_cm_ to *V_ref_*, and *R*_N1_ is changed from *V*_cm_ to *gnd*. The output voltage is found to be

(2) $$\left\{ \begin{array}{l} V_{P}\left(2\right)=V_{ip}+\left[1-2D_{1}\right]\cdot \frac{V_{ref}}{4}\\ V_{N}\left(2\right)=V_{in}+\left[2D_{1}-1\right]\cdot \frac{V_{ref}}{4} \end{array}\right.$$

The comparator compares *V_P_*(2) with *V_N_*(2) and obtains *D*_2_.

In the 3rd comparison, if *D*_2_ is 1, the reference voltage of *R*_P4_ is changed from *V*_cm_ to *gnd*, and *R*_N4_ is changed from *V*_cm_ to *V_ref_*. If *D*_4_ is 0, the reference voltage of *R*_P4_ is changed from *V*_cm_ to *V_ref_*, and *R*_N4_ is changed from *V*_cm_ to *gnd*. The output voltage is found to be

(3) $$\left\{ \begin{array}{l} V_{P}\left(3\right)=V_{ip}+\left[1-2D_{1}\right]\cdot \frac{V_{ref}}{4}+\left[1-2D_{2}\right]\cdot \frac{V_{ref}}{8}\\ V_{N}\left(3\right)=V_{in}+\left[2D_{1}-1\right]\cdot \frac{V_{ref}}{4}+\left[2D_{2}-1\right]\cdot \frac{V_{ref}}{8} \end{array}\right.$$

The comparator compares *V_P_*(3) with *V_N_*(3) and obtains *D*_3_.

From the 3rd comparison to the 8th comparison, the DAC performs *V*_cm_-based switching scheme [11].

#### 2.1.2. Proposed 9-Bit Mode Switching Scheme

**Figure 3:**
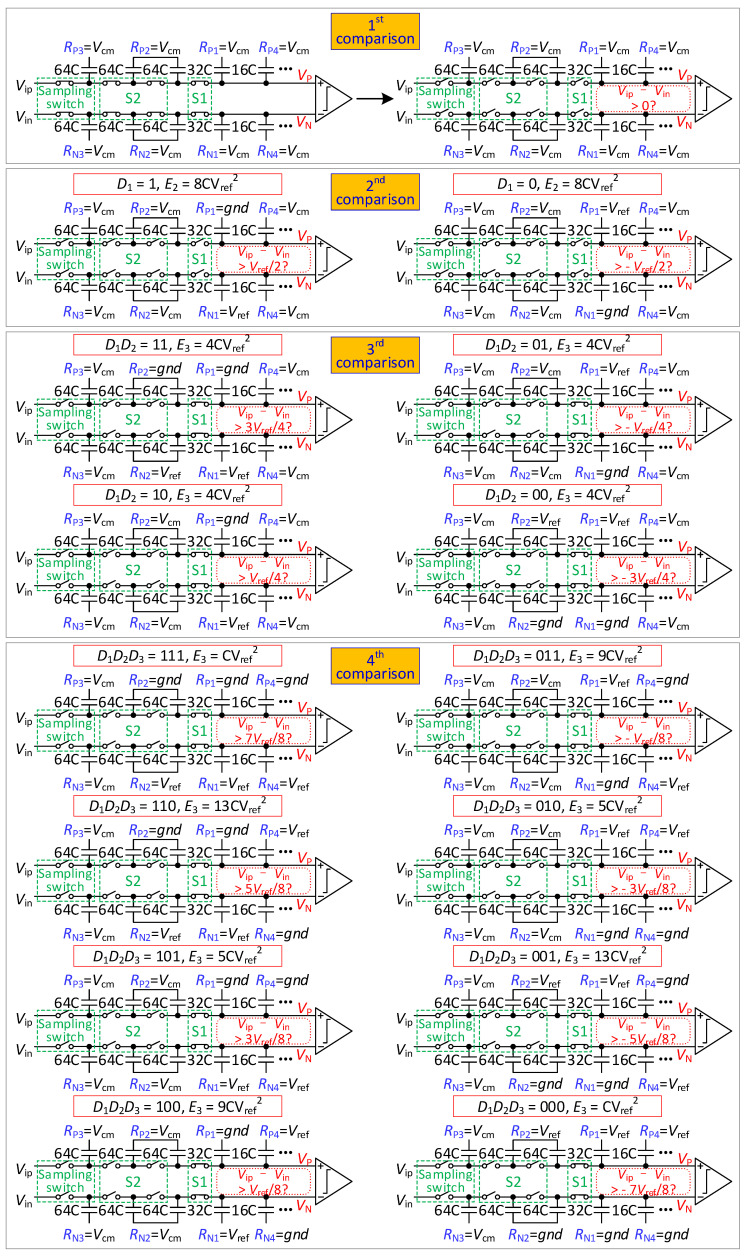
Proposed 9-bit mode switching scheme.

The steps of the conversion process of 9-bit mode are illustrated in Figure 3.

From the sampling phase to the 2nd comparison, a conversion process of 9-bit mode is the same as that of 8-bit mode.

In the 3rd comparison, S1 is closed. If *D*_1_*D*_2_ is 11, the reference voltage of *R*_P2_ is changed from *V*_cm_ to *gnd*, and *R*_N2_ is changed from *V*_cm_ to *V_ref_.* If *D*_1_*D*_2_ is 00, the reference voltage of *R*_P2_ is changed from *V*_cm_ to *V_ref_*, and *R*_N2_ is changed from *V*_cm_ to *gnd*. If *D*_1_*D*_2_ is 01 or 10, the reference voltage of *R*_P2_ and *R*_N2_ remains unchanged. The output voltage is shown in equation 3. The comparator compares *V_P_*(3) with *V_N_*(3) and obtains *D*_3_.

In the 4th comparison, if *D*_3_ is 1, the reference voltage of *R*_P4_ is changed from *V*_cm_ to *gnd*, and *R*_N4_ is changed from *V*_cm_ to *V_ref_*. If *D*_3_ is 0, the reference voltage of *R*_P4_ is changed from *V*_cm_ to *V_ref_*, and *R*_N4_ is changed from *V*_cm_ to *gnd*. The output voltage is found to be

(4) $$\left\{ \begin{array}{l} V_{P}\left(4\right)=V_{ip}+\left[1-2D_{1}\right]\cdot \frac{V_{ref}}{4}+\left[1-2D_{2}\right]\cdot \frac{V_{ref}}{8}+\left[1-2D_{3}\right]\cdot \frac{V_{ref}}{16}\\ V_{N}\left(4\right)=V_{in}+\left[2D_{1}-1\right]\cdot \frac{V_{ref}}{4}+\left[2D_{2}-1\right]\cdot \frac{V_{ref}}{8}+\left[2D_{3}-1\right]\cdot \frac{V_{ref}}{16} \end{array}\right.$$

The comparator compares *V_P_*(4) with *V_N_*(4) and obtains *D*_4_.

From the 4th comparison to the 9th comparison, the DAC performs a *V*_cm_-based switching scheme [11].

#### 2.1.3. Proposed 10-Bit Mode Switching Scheme

**Figure 4:**
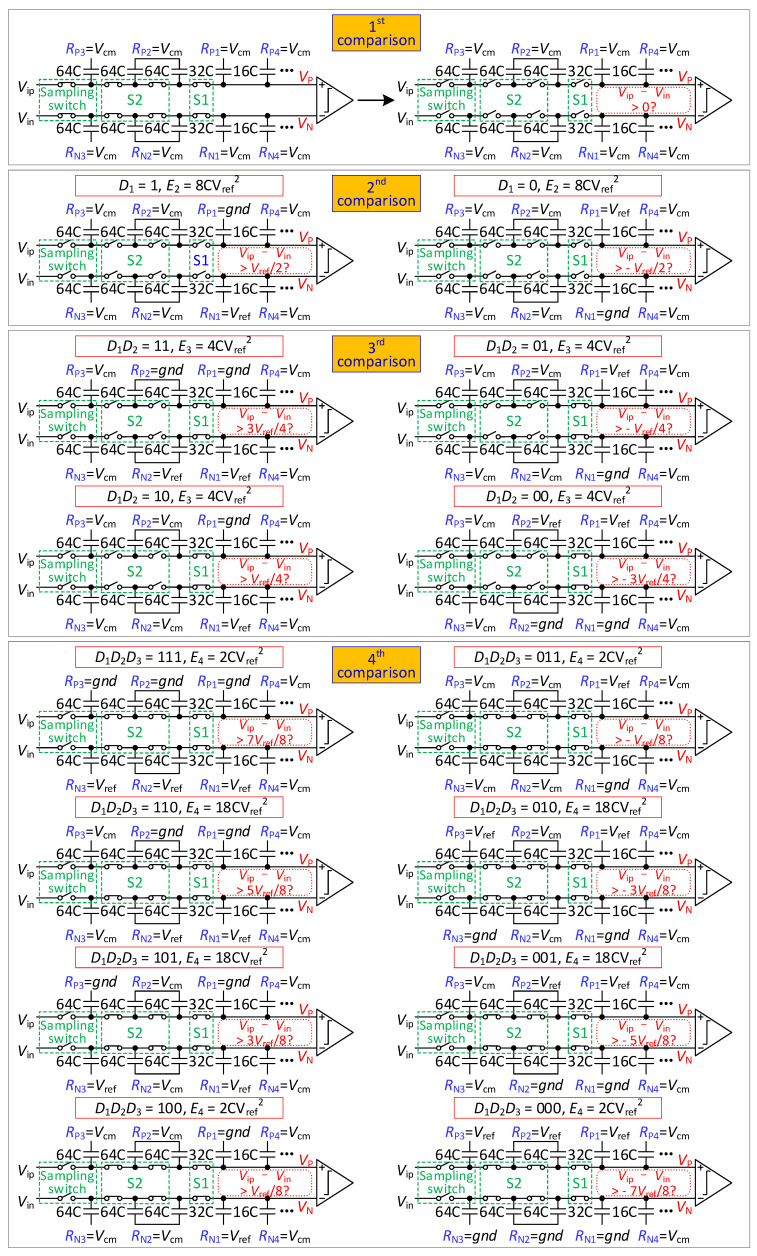
The 1st to 4th comparison diagram of the proposed 10-bit mode switching scheme.

**Figure 5:**
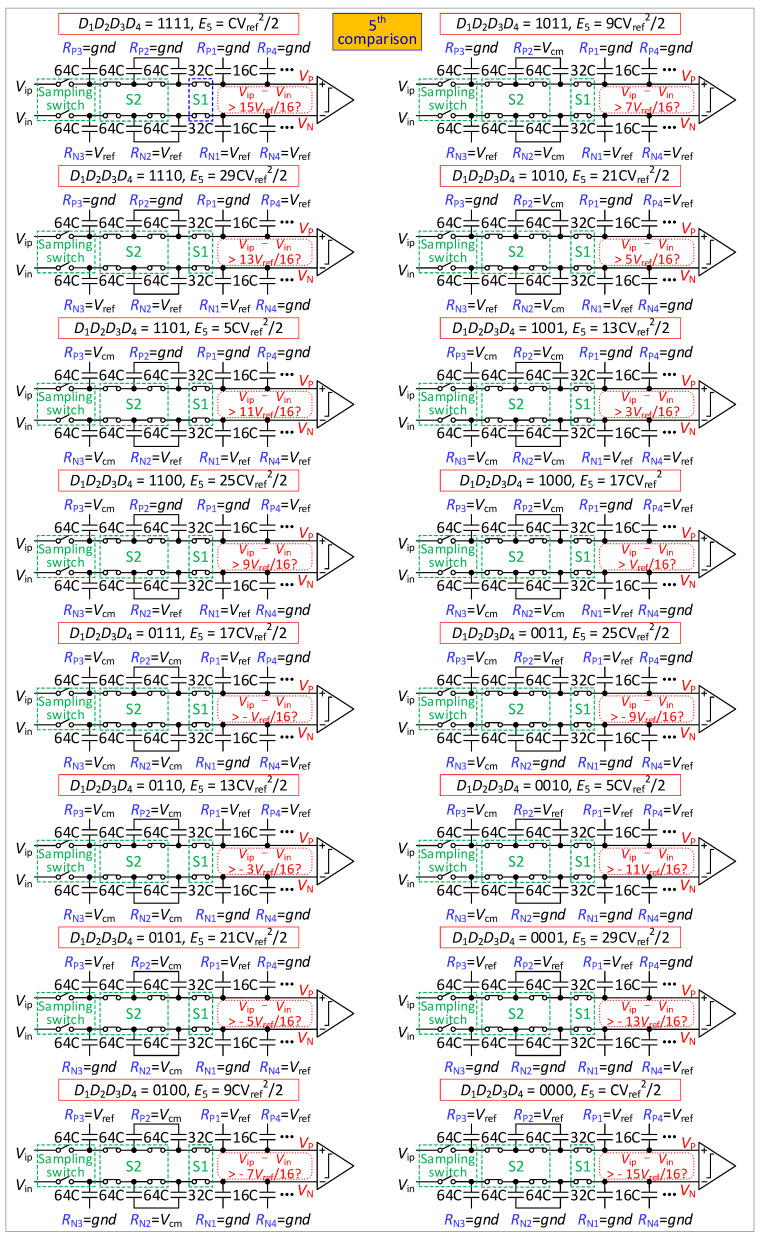
The 5th comparison diagram of the proposed 10-bit mode switching scheme.

The steps of the conversion process of 10-bit are illustrated in Figure 4 and Figure 5.

From the sampling phase to the 3rd comparison, the conversion process of 10-bit mode is the same as that of 9-bit mode.

In the 4th comparison, S2 is closed. If *D*_1_*D*_3_ is 11, the reference voltage of *R*_P3_ is changed from *V*_cm_ to *gnd*, and *R*_N3_ is changed from *V*_cm_ to *V_ref_*. If *D*_1_*D*_3_ is 00, the reference voltage of *R*_P3_ is changed from *V*_cm_ to *V_ref_*, and *R*_N3_ is changed from *V*_cm_ to *gnd*. If *D*_1_*D*_3_ is 01 or 10, the reference voltage of *R*_P3_ and *R*_N3_ remains unchanged. The output voltage is found to be

(5) $$\left\{ \begin{array}{l} V_{P}\left(4\right)=V_{ip}+\left[1-2D_{1}\right]\cdot \frac{V_{ref}}{4}+\left[1-2D_{2}\right]\cdot \frac{V_{ref}}{8}+\left[1-2D_{3}\right]\cdot \frac{V_{ref}}{16}\\ V_{N}\left(4\right)=V_{in}+\left[2D_{1}-1\right]\cdot \frac{V_{ref}}{4}+\left[2D_{2}-1\right]\cdot \frac{V_{ref}}{8}+\left[2D_{3}-1\right]\cdot \frac{V_{ref}}{16} \end{array}\right.$$

The comparator compares *V_P_*(4) with *V_N_*(4) and obtains *D*_4_.

In the 5th comparison, if *D*_4_ is 1, the reference voltage of *R*_P4_ is changed from *V*_cm_ to *gnd*, and *R*_N4_ is changed from *V*_cm_ to *V_ref_*. If *D*_4_ is 0, the reference voltage of *R*_P4_ is changed from *V*_cm_ to *V_ref_*, and *R*_N4_ is changed from *V*_cm_ to *gnd*. The output voltage is found to be

(6) $$\left\{ \begin{array}{l} V_{P}\left(5\right)=V_{ip}+\sum_{j=1}^{4}\left[1-2D_{j}\right]\cdot \frac{V_{ref}}{2^{j+1}}\\ V_{N}\left(5\right)=V_{in}+\sum_{j=1}^{4}\left[2D_{j}-1\right]\cdot \frac{V_{ref}}{2^{j+1}} \end{array}\right.$$

The comparator compares *V_P_*(5) with *V_N_*(5) and obtains *D*_5_.

From the 5th comparison to the 10th comparison, the DAC performs a *V*_cm_-based switching scheme [11]. Because the large capacitor does not participate in the first and second comparisons, it is more energy-efficient than a *V*_cm_-based switching scheme.

**Figure 6:**
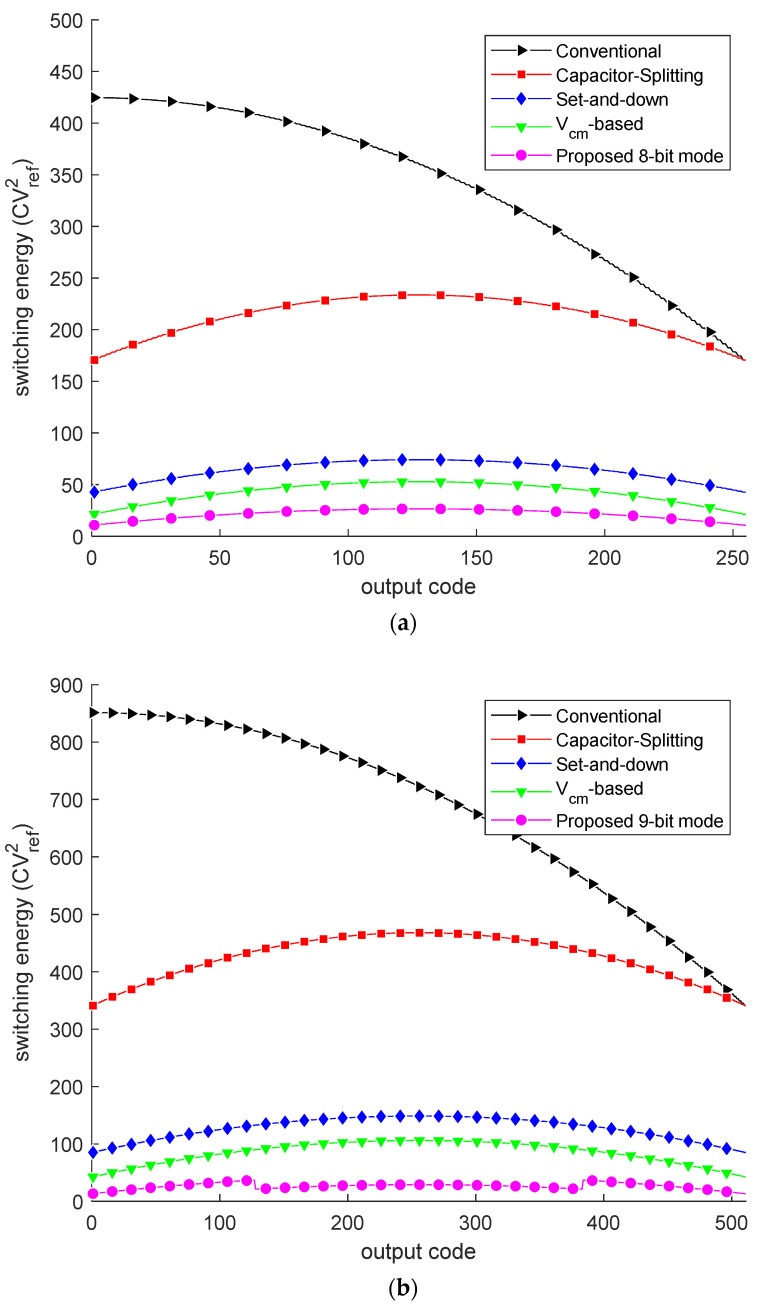

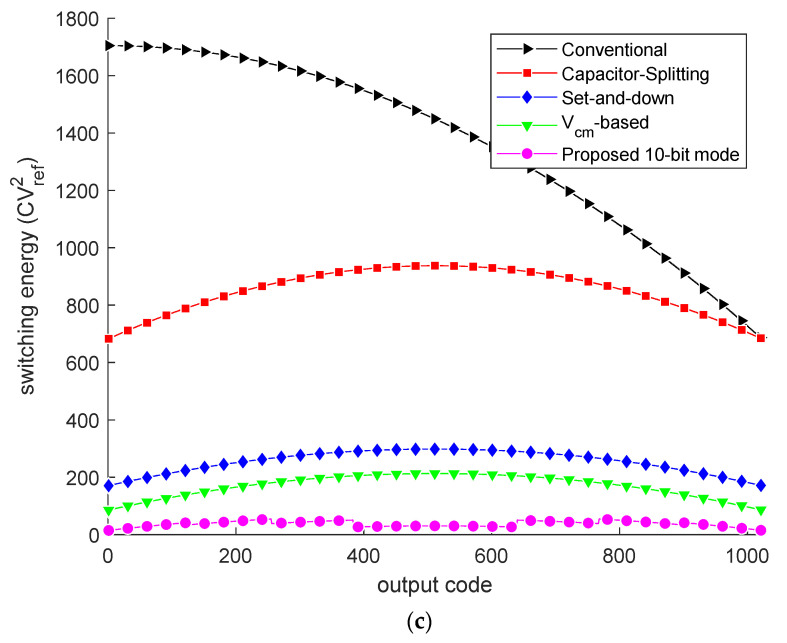
Switching energy against output codes: (**a**) 8-bit; (**b**) 9-bit; (**c**) 10-bit. The black [12], red [9], blue [10], green [11] and magenta curves in the three figures are switching energy.

**Figure 7:**
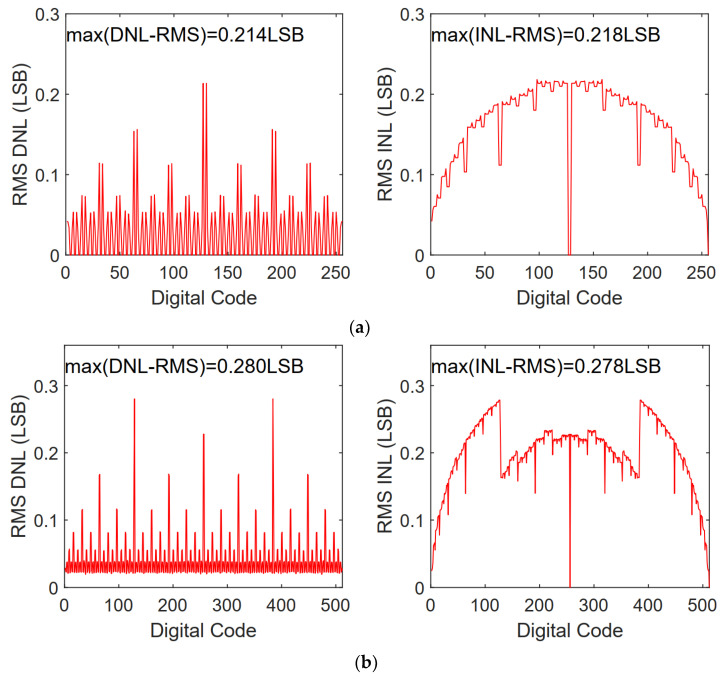

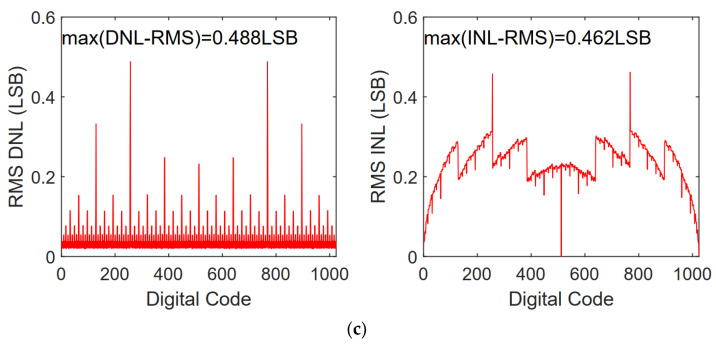
DNL and INL versus output code of the proposed switching scheme: (**a**) 8-bit mode; (**b**) 9-bit mode; (**c**) 10-bit mode.

Based on the switching energy calculation method in [9], the switching energy of different switching schemes is calculated and shown in Figure 6 and Table 5. Figure 6 shows switching energy at each output code for different switching schemes. Benefiting from the resolution-reconfigurable technology, the proposed switching scheme has lower switching energy in middle output codes for the 9-bit and 10-bit modes. As shown in Table 5 for the proposed switching scheme, the more bits, the more energy is saved; the scheme saves 96.1% and 97.3% of switching energy in 9-bit and 10-bit modes, respectively. Figure 7 presents the 500-run Monte Carlo simulation results of the proposed DAC switching scheme with unit capacitor mismatch of σu/Cu = 2%. The RMS DNL and the RMS INL of the proposed DAC switching scheme are 0.214/0.280/0.488 LSB and 0.218/0.278/0.462 LSB, corresponding to the 8/9/10-bit mode, respectively.

### 2.2. Sampling Switch

**Figure 8:**
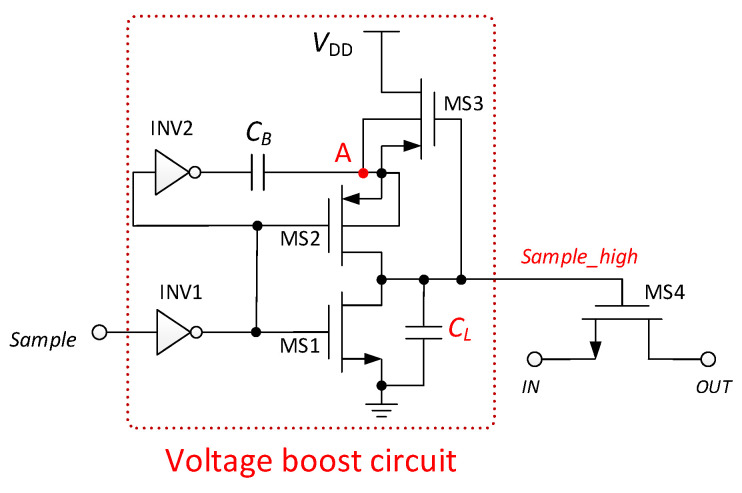
Voltage bootstrap sampling circuit.

In order to reduce the on-resistance of the sampling switch and reduce the sampling error, the sampling switch adopts a bootstrap switch circuit [13]. The voltage bootstrap sampling circuit is shown in Figure 8. When the “*Sample*” voltage is low, the transistors MS1 and MS3 are turned on, MS2 and MS4 are turned off, the voltage of node A is charged to *V_DD_*, and the “*Sample_high*” voltage is low. When the “*Sample*” voltage becomes high, the transistor MS1 is turned off, MS2 is turned on, MS3 is turned off, and MS4 is turned on; the voltage of node A is boosted to 2*V_DD_* by the capacitor *C*_B_, and the “*Sample_high*” voltage starts to increase. The “*Sample_high*” voltage boost expression is as follows:

(7) $$V_{sample\_high}=2V_{DD}\frac{C_{B}}{C_{B}+C_{L}}$$

*C_L_* is the parasitic capacitance. If *C_B_* is much larger than *C_L_*, the “*Sample_high*” voltage is raised to about twice *V_DD_*.

**Figure 9:**
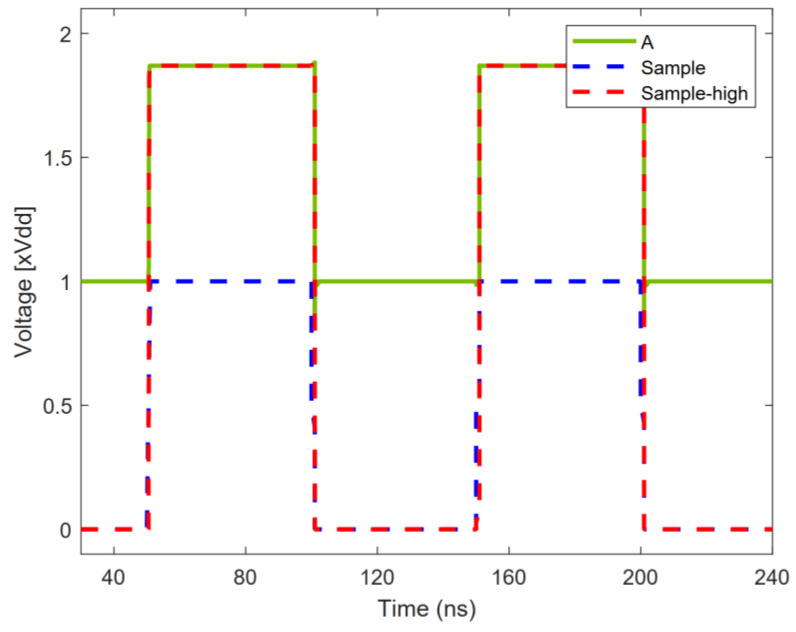
Transient simulation of sampling switch.

**Figure 10:**
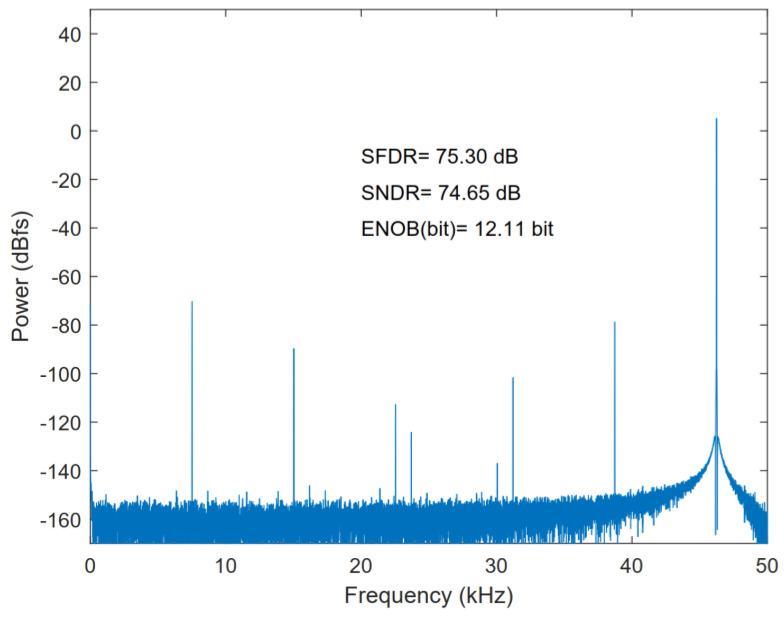
FFT of sampling switch.

Figure 9 illustrates the voltage bootstrap of the sampling switch. The FFT of the sampling switch is shown in Figure 10. The spurious-free dynamic range (SFDR) and the signal-to-noise-plus-distortion ratio (SNDR) of the sampling switch are 75.30 and 74.65 dB, respectively.

### 2.3. Comparator

**Figure 11:**
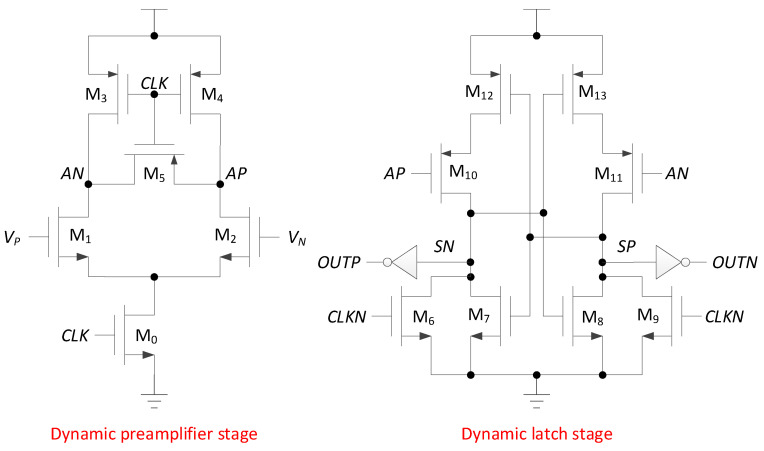
Two-stage fully dynamic comparator.

To decrease the power consumption of the comparator, a two-stage full dynamic comparator [14] is used. Figure 11 shows the schematic diagram of the comparator. The first stage is the dynamic preamplifier stage, and *V_P_* and *V_N_* are the output signals of the capacitor array DAC, connected to the differential input of the comparator. *AN* and *AP* are differential outputs of the dynamic preamplifier stage. The second stage is the dynamic latch stage, which is responsible for the two-stage amplification and the result latch, and *OUTP* and *OUTN* are the comparison results.

**Figure 12:**
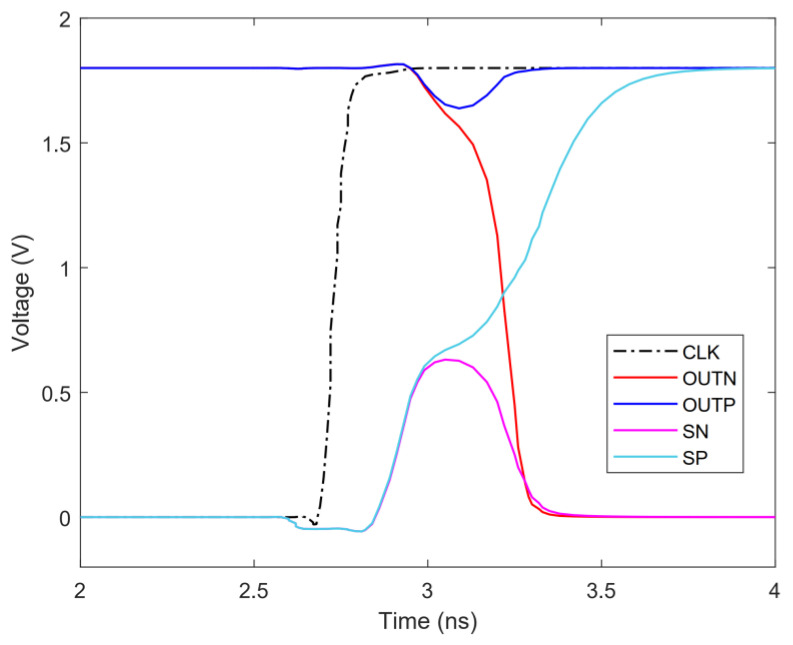
Transient simulation of comparator.

**Figure 13:**
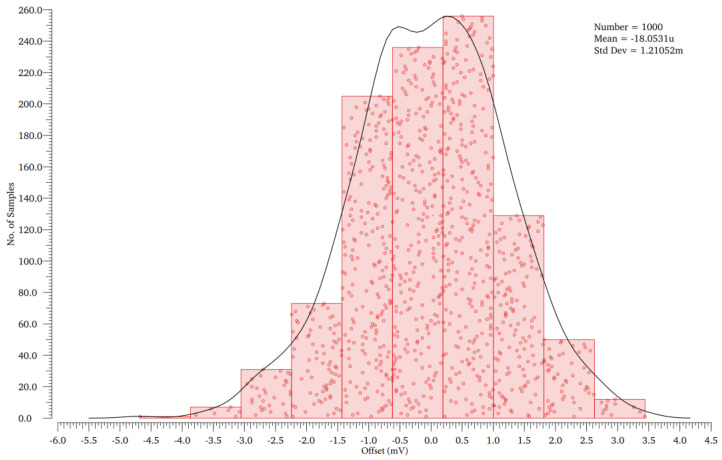
Monte Carlo simulation of the offset voltage for 1000 points.

When CLK is low (CLKN is high), M0 is off, M3 and M4 are on, *AN* and *AP* are charged to high (M5 makes *AP* and *AN* charge balance), M6 and M9 are on, *OUTP* and *OUTN* are pulled up to high. When *CLK* is high (*CLKN* is low), M3, M4 and M5 are turned off, M0 is turned on, *AN* and *AP* are discharged through M1 and M2, respectively, and the speed of discharge depends on the voltage of *V_P_* and *V_N_* (if *V_P_* > *V_N_*, then *AP* > *AN*; if *V_P_* < *V_N_*, then *AP* < *AN*). At this time, M6 and M9 of the dynamic latch stage are turned off, *SP* and *SN* are charged by M10 and M11, and the charging speed depends on the voltage of AP and *AN* (if *AP* > *AN*, then *SP* > *SN*; if *AP* < *AN*, then *SP* < *SN*). If *SP* or *SN* rises to the threshold voltage, M7 or M8 will be turned on, positive feedback will start to work, and one of *SP* and *SN* quickly rises to high and the other pulls low to complete the latching of the comparison result. Since the dynamic comparator does not form a power-to-ground path when operating, the comparator has only dynamic power. The transient simulation of the comparator is shown in Figure 12. Figure 13 shows the Monte Carlo simulations that are performed to observe the effect of mismatches and process variations on offset voltage. Offset voltage has a mean value of −18.053 µV with the standard deviation (SD) of 1.21052 mV.

### 2.4. SAR Control Logic

**Figure 14:**
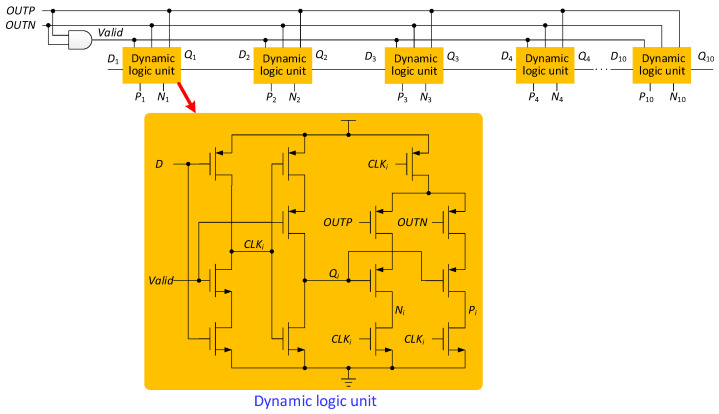
SAR based on dynamic logic.

**Figure 15:**
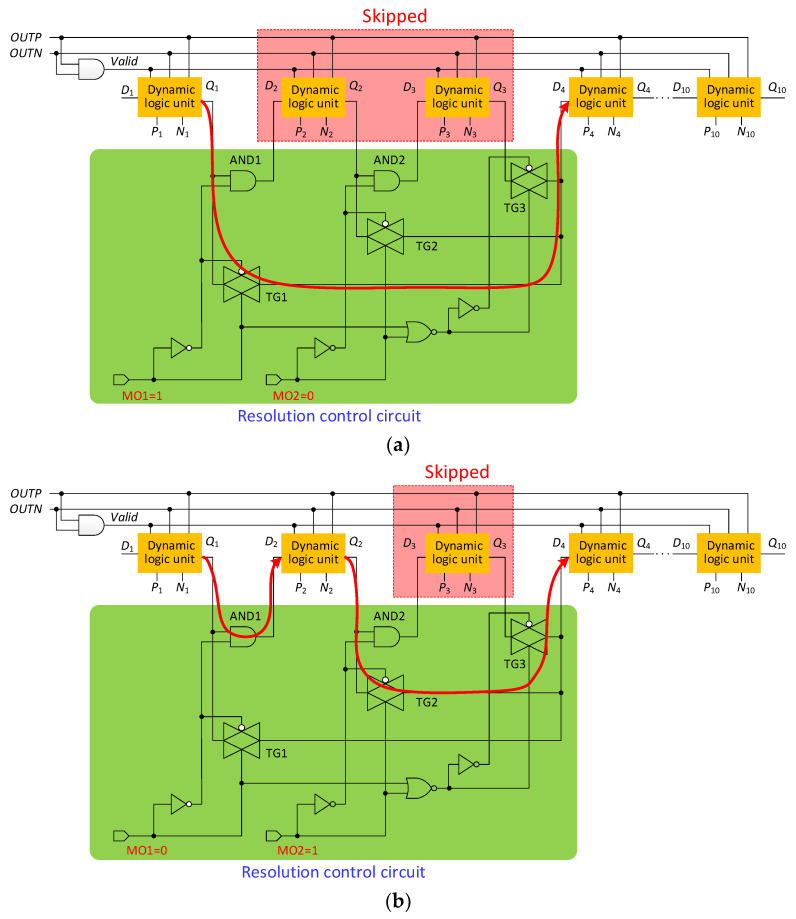

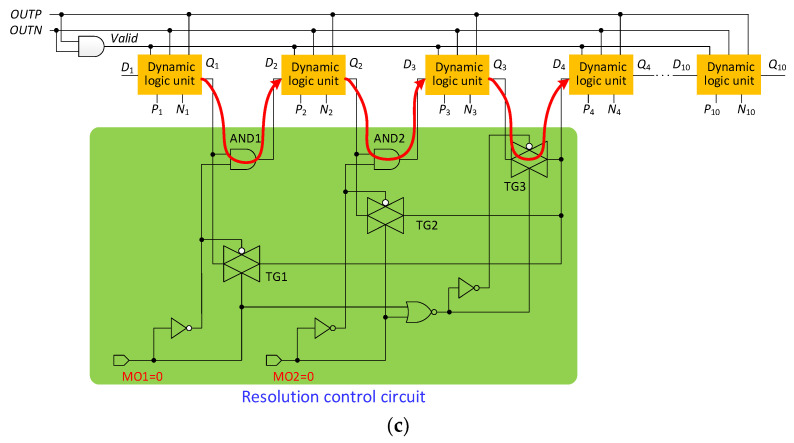
SAR control logic with bit control circuit: (**a**) 8-bit mode; (**b**) 9-bit mode; (**c**) 10-bit mode.

To improve the performance of SAR logic, a dynamic logic unit is used [15]. As shown in Figure 14, the SAR logic is composed of dynamic logic units, one by one. The dynamic logic unit has both a shift function and a function of storing comparison results, which saves many transistors compared to conventional shift registers. The 10-bit SAR logic has 10 dynamic logic units. When the SAR ADC is operating in 8-bit or 9-bit resolutions, in order to save power, two or one dynamic logic units need to be skipped, respectively. As shown in Figure 15, a resolution control circuit is added to SAR logic. The resolution of SAR logic is controlled by MO1 and MO2. Different resolutions will form different circuit paths. The resolution settings are shown in Table 6.

Figure 15a shows the 8-bit mode work diagram of SAR control logic. When MO1 = 1 and MO2 = 0, the circuit is set to 8-bit operating mode, the transmission gate TG1 is turned on, the AND gates AND1 and AND2 are turned off, and the transmission gates TG2 and TG3 are turned off. Then, the output *Q*_1_ of the first dynamic logic unit is directly connected to the input *D*_4_ of the fourth dynamic logic unit; the second and third dynamic logic units are skipped.

Figure 15b shows the 9-bit mode work diagram of SAR control logic. When MO1 = 0 and MO2 = 1, the circuit is set to 9-bit operating mode, AND1 and TG2 are turned on, and AND2, TG1, and TG3 are turned off. Then, the output *Q*_2_ of the second dynamic logic unit is directly connected to the input *D*_4_ of the fourth dynamic logic unit; the third dynamic logic unit is skipped.

Figure 15c shows the 10-bit mode work diagram of SAR control logic. When MO1 = 0 and MO2 = 0, the circuit is set to 10-bit operating mode, AND1, AND2 and TG3 are turned on, and TG1 and TG2 are turned off. In this case, no dynamic logic cells are skipped.

## 3. Results

**Figure 16:**
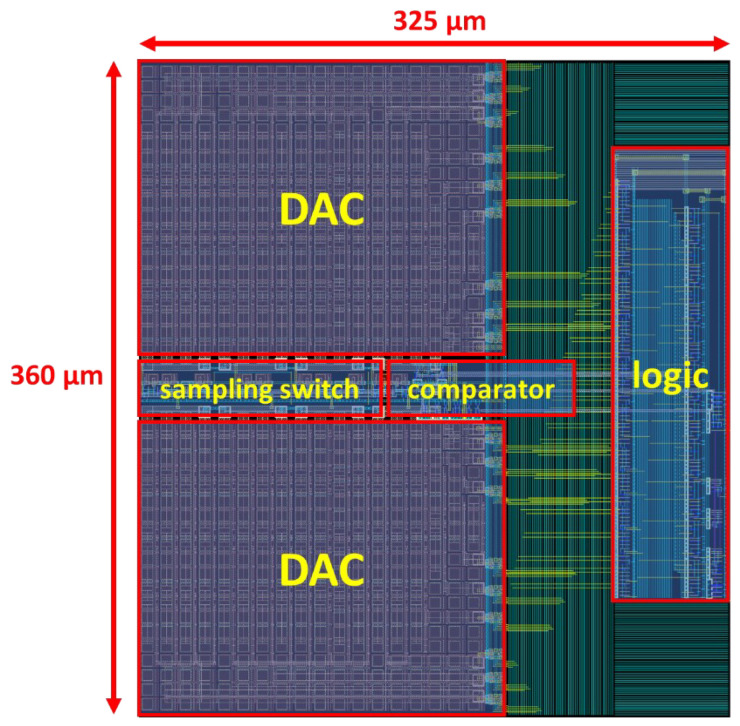
Layout of the ADC.

**Figure 17:**
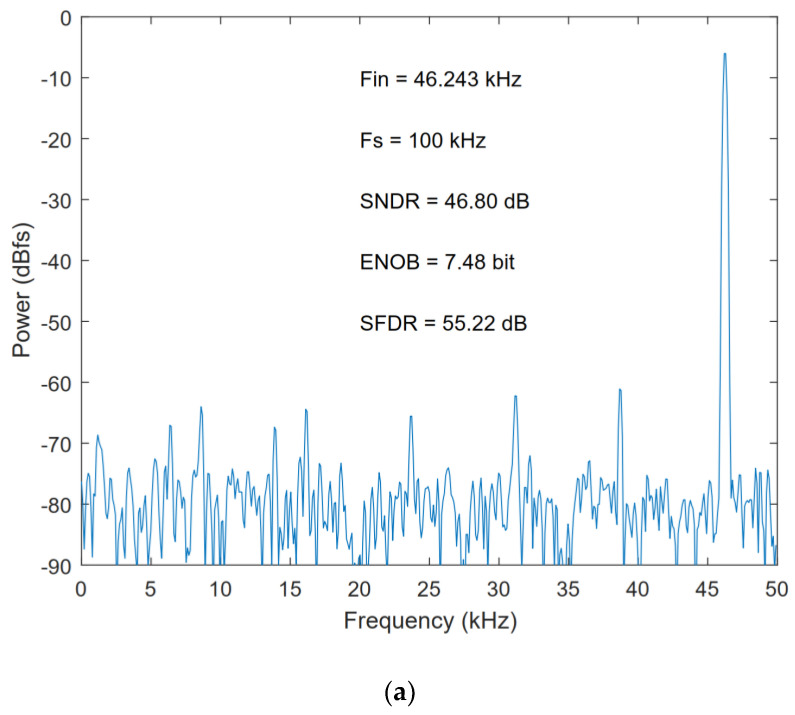

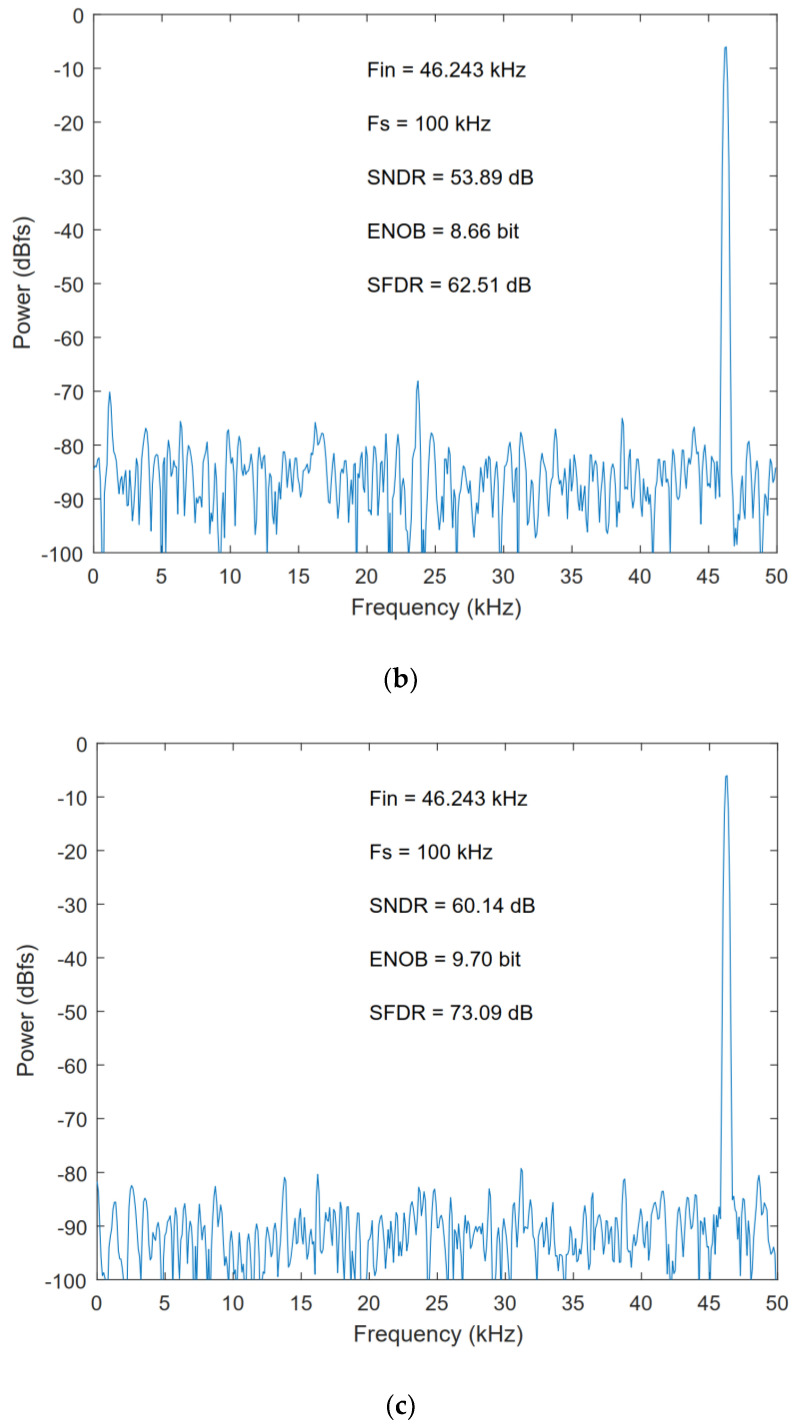
FFT of ADC: (**a**) 8-bit mode; (**b**) 9-bit mode; (**c**) 10-bit mode.

**Figure 18:**
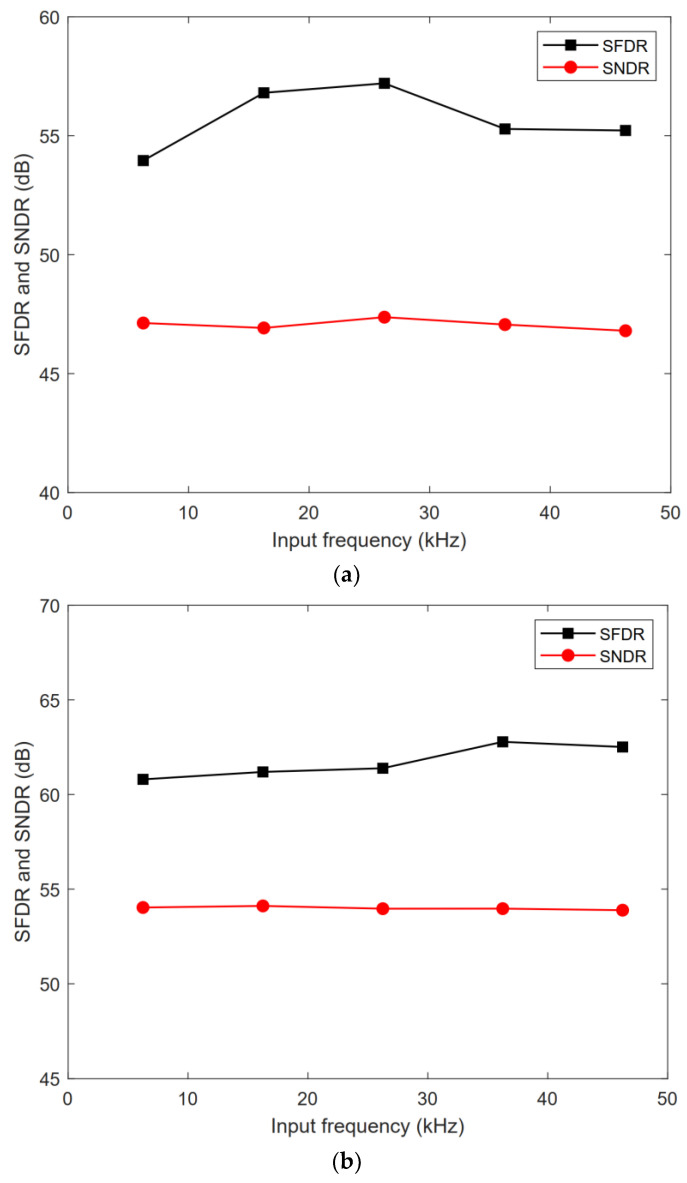

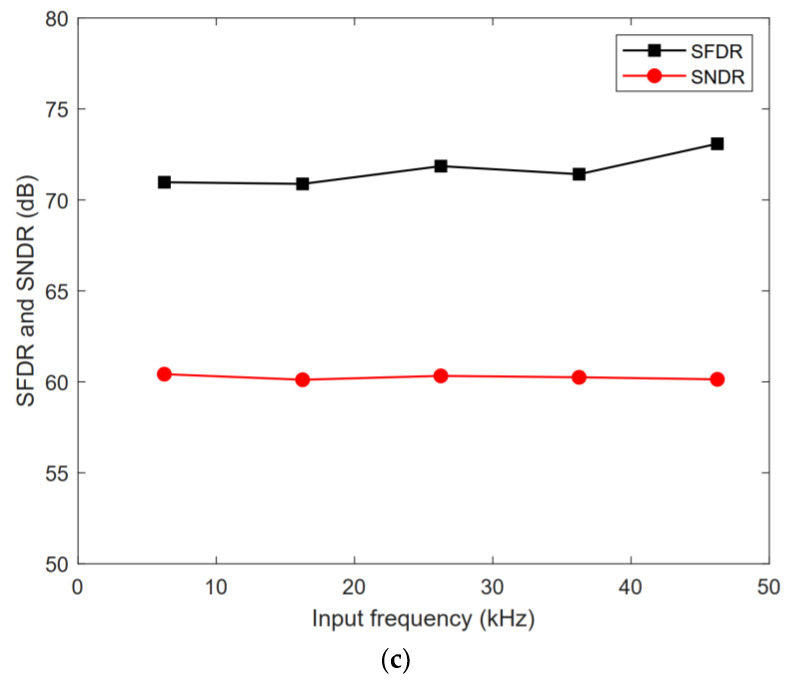
SNDR/SFDR with various input frequencies: (**a**) 8-bit mode; (**b**) 9-bit mode; (**c**) 10-bit mode.

**Figure 19:**
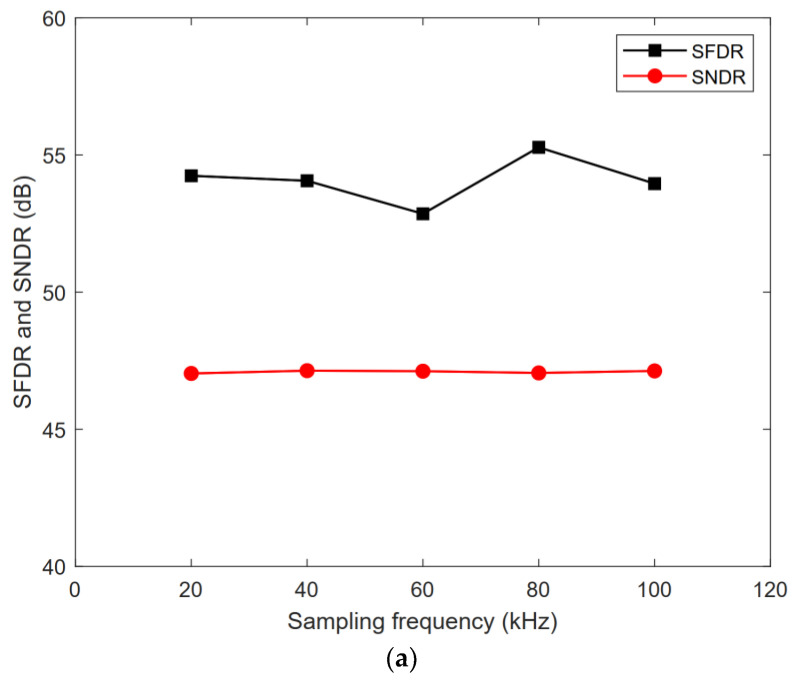

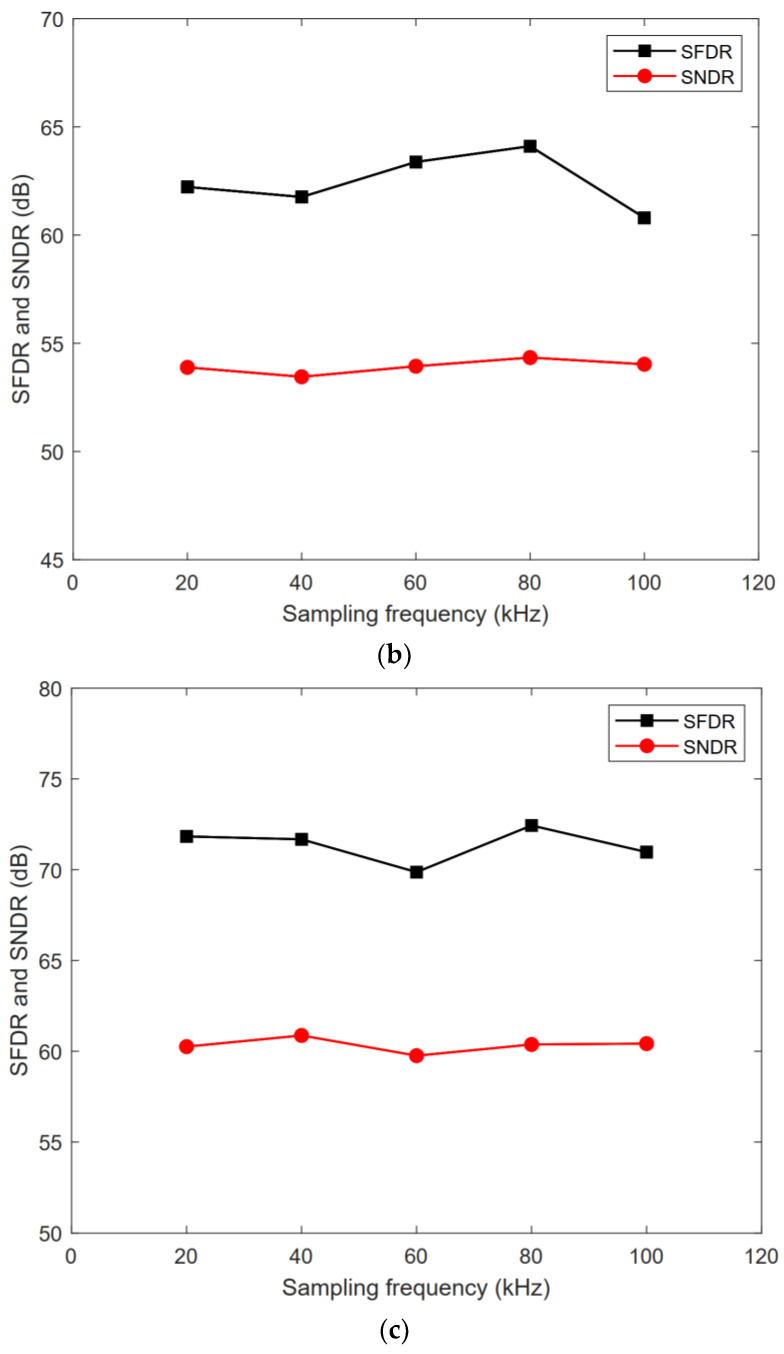
SNDR/SFDR with various sampling frequencies: (**a**) 8-bit mode; (**b**) 9-bit mode; (**c**) 10-bit mode.

**Figure 20:**
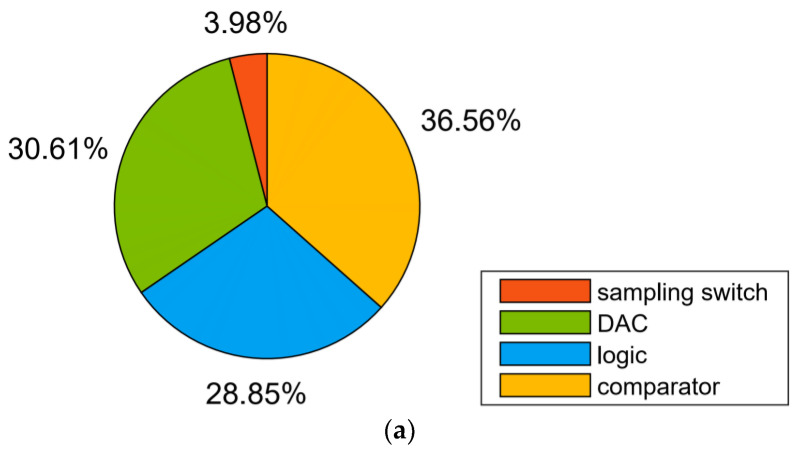

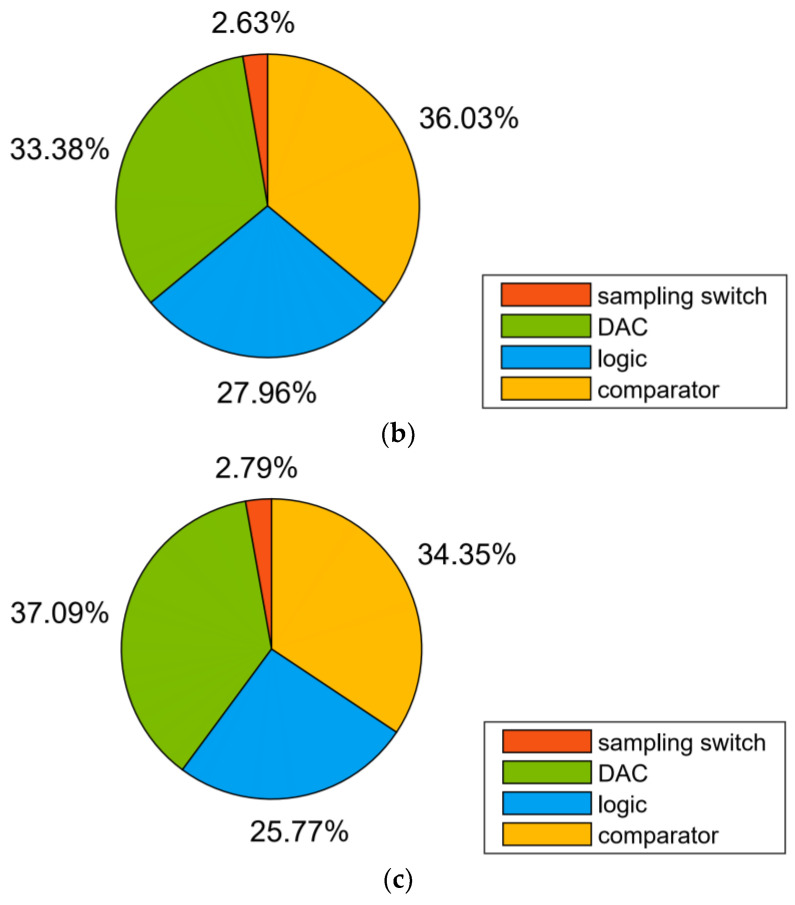
Power breakdown of the ADC: (**a**) 8-bit mode; (**b**) 9-bit mode; (**c**) 10-bit mode.

The proposed ADC was designed and post-simulated using 180 nm CMOS technology. Figure 16 shows the layout of the ADC with a total area of 360 μm × 325 μm. Figure 17 shows the FFT spectrum of the proposed ADC in 8-bit, 9-bit, and 10-bit modes with the 1.8 V full swing inputs at 46.243 kHz and the sampling rate at 100 kS/s; the ADC achieves the 46.80/53.89/60.14 dB signal-to-noise and distortion ratio (SNDR) and 55.22/62.51/73.09 dB spurious-free dynamic range (SFDR), respectively. Figure 18 shows the SNDR and SFDR of the proposed SAR ADC with respect to the input frequency. Figure 19 shows the SNDR and SFDR of the proposed SAR ADC with respect to the sampling frequency. The proposed ADC consumes 0.81/0.91/1.01 μW corresponding to the 8/9/10-bit mode, respectively. Figure 20 shows the power breakdown of the ADC.

Table 7 compares the proposed ADC with other ADCs [16,17,18,19]. As shown in Table 7, the performance of the proposed ADC is still competitive when it is implemented in 0.18 μm 1.8 V CMOS process. The Figure-of-Merit (FoM) was calculated from the following equation:

(8) $$FoM=\frac{Power}{2^{ENOB}\times f_{samping}}$$

## 4. Conclusions

In this paper, a reconfigurable 8–10-bit SAR ADC with an energy-efficient DAC switching scheme for biosensor applications is presented. Several techniques are used to enable the reconfiguration. Simulated with a 180 nm CMOS process and 100 kS/s sampling rate, the ADC achieves the 46.80/53.89/60.14 dB SNDR, the 55.22/62.51/73.09 dB SFDR, and the 0.81/0.91/1.01 μW power consumption in 8/9/10-bit mode, respectively.

## Figures and Tables

**Table 1:** The frequency range of various biomedical signals.

Biomedical Signals	Frequency Range
Electroencephalogram (EEG)	1–150 Hz
Electromyogram (EMG)	25–1 kHz
Electrocardiogram (ECG)	0.5–250 Hz
Local Field Potential (LFP)	0.5–200 Hz
Action Potential (AP)	100–7 kHz

**Table 2:** Resolutions of ADC are adjusted by S1 and S2.

Resolution	Phase				
	Sampling	Comparison			
		1st	2nd	3rd	4th
8-bit mode	S1:close	S1:open	S1:open	S1:open	S1:open
	S2:close	S2:open	S2:open	S2:open	S2:open
9-bit mode	S1:close	S1:open	S1:open	S1:close	S1:close
	S2:close	S2:open	S2:open	S2:open	S2:open
10-bit mode	S1:close	S1:open	S1:open	S1:close	S1:close
	S2:close	S2:open	S2:open	S2:open	S2:close

**Table 3:** *R*_P2_ and *R*_N2_ for each phase of 9-bit and 10-bit mode.

*D*_1_ *D*_2_	Phase			
	Sampling	Comparison		
		1st	2nd	3rd
00	*R*_P2_ = *V*_cm_	*R*_P2_ = *V*_cm_	*R*_P2_ = *V*_cm_	*R*_P2_ = *V*_ref_
	*R*_N2_ = *V*_cm_	*R*_N2_ = *V*_cm_	*R*_N2_ = *V*_cm_	*R*_N2_ = *gnd*
01	*R*_P2_ = *V*_cm_	*R*_P2_ = *V*_cm_	*R*_P2_ = *V*_cm_	*R*_P2_ = *V*_cm_
	*R*_N2_ = *V*_cm_	*R*_N2_ = *V*_cm_	*R*_N2_ = *V*_cm_	*R*_N2_ = *V*_cm_
10	*R*_P2_ = *V*_cm_	*R*_P2_ = *V*_cm_	*R*_P2_ = *V*_cm_	*R*_P2_ = *V*_cm_
	*R*_N2_ = *V*_cm_	*R*_N2_ = *V*_cm_	*R*_N2_ = *V*_cm_	*R*_N2_ = *V*_cm_
11	*R*_P2_ = *V*_cm_	*R*_P2_ = *V*_cm_	*R*_P2_ = *V*_cm_	*R*_P2_ = *gnd*
	*R*_N2_ = *V*_cm_	*R*_N2_ = *V*_cm_	*R*_N2_ = *V*_cm_	*R*_N2_ = *V*_ref_

**Table 4:** *R*_P3_ and *R*_N3_ for each phase of 10-bit mode.

*D*_1_ *D*_3_	Phase				
	Sampling	Comparison			
		1st	2nd	3rd	4th
00	*R*_P3_ = *V*_cm_	*R*_P3_ = *V*_cm_	*R*_P3_ = *V*_cm_	*R*_P3_ = *V*_cm_	*R*_P3_ = *V*_ref_
	*R*_N3_ = *V*_cm_	*R*_N3_ = *V*_cm_	*R*_N3_ = *V*_cm_	*R*_N3_ = *V*_cm_	*R*_N3_ = *gnd*
01	*R*_P3_ = *V*_cm_	*R*_P3_ = *V*_cm_	*R*_P3_ = *V*_cm_	*R*_P3_ = *V*_cm_	*R*_P3_ = *V*_cm_
	*R*_N3_ = *V*_cm_	*R*_N3_ = *V*_cm_	*R*_N3_ = *V*_cm_	*R*_N3_ = *V*_cm_	*R*_N3_ = *V*_cm_
10	*R*_P3_ = *V*_cm_	*R*_P3_ = *V*_cm_	*R*_P3_ = *V*_cm_	*R*_P3_ = *V*_cm_	*R*_P3_ = *V*_cm_
	*R*_N3_ = *V*_cm_	*R*_N3_ = *V*_cm_	*R*_N3_ = *V*_cm_	*R*_N3_ = *V*_cm_	*R*_N3_ = *V*_cm_
11	*R*_P3_ = *V*_cm_	*R*_P3_ = *V*_cm_	*R*_P3_ = *V*_cm_	*R*_P3_ = *V*_cm_	*R*_P3_ = *gnd*
	*R*_N3_ = *V*_cm_	*R*_N3_ = *V*_cm_	*R*_N3_ = *V*_cm_	*R*_N3_ = *V*_cm_	*R*_N3_ = *V*_ref_

**Table 5:** Comparison of several switching schemes for SAR ADC.

Switching Scheme	$\mathrm{Average}\;\mathrm{Switching}\;\mathrm{Energy}\;(CV_{ref}^{2})$			Energy Saving		
	8-Bit	9-Bit	10-Bit	8-Bit	9-Bit	10-Bit
Conventional [12]	339.3	680.7	1363.3	Reference	Reference	Reference
Split capacitor [9]	212.3	425.7	852.3	37.4%	37.4%	37.4%
Set-and-down [10]	63.5	127.5	255.5	81.3%	81.3%	81.3%
V_cm_-based [11]	42.2	84.8	170.2	87.5%	87.5%	87.5%
Proposed	21.2	26.5	37.1	93.8%	96.1%	97.3%

**Table 6:** The resolution settings of SAR logic.

MO1	MO2	Bit Mode
1	0	8-bit mode
0	1	9-bit mode
0	0	10-bit mode

**Table 7:** Performance comparison.

Article Title	[16]	[17]	[18] *	[19]	This Work *		
Technology (nm)	180	180	180	65	180	180	180
Supply Voltage (V)	0.9	1.0	1.8	1.2	1.8	1.8	1.8
Resolution (bit)	9	10	10	10	8	9	10
Sampling Rate (KS/s)	100	100	25	25	100	100	100
Power (μW)	1.33	1.72	2.83	0.84	0.81	0.91	1.01
ENOB (bit)	8.02	9.48	9.77	9.59	7.48	8.66	9.70
FoM (fJ/conv.-step)	51.3	24.1	129	43.60	45.37	22.5	12.1

* Simulated results.

## Data Availability

Not applicable.
